# Space-time finite element methods stabilized using bubble function spaces

**DOI:** 10.1080/00036811.2018.1522630

**Published:** 2018-09-24

**Authors:** Ioannis Toulopoulos

**Affiliations:** a Johann Radon Institute for Computational and Applied Mathematics (RICAM), Austrian Academy of Sciences, Linz, Austria; b AC2T research GmbH, Austrian Excellence Center for Tribology, Wiener Neustadt, Austria

**Keywords:** Parabolic initial-boundary value problems, space-time finite element methods, bubble stabilization, optimal convergence rates

## Abstract

In this paper, a stabilized space-time finite element method for solving linear parabolic evolution problems is analyzed. The proposed method is developed on a base of a space-time variational setting, that helps on the simultaneous and unified discretization in space and in time by finite element techniques. Stabilization terms are constructed by means of classical bubble spaces. Stability of the discrete problem with respect to an associated mesh dependent norm is proved, and a priori discretization error estimates are presented. Numerical examples confirm the theoretical estimates.

## 1. Introduction

Parabolic evolution equations are used to describe numerous physical phenomena, as for example heat transfer. The traditional methods for parabolic problems usually apply a separate method for the time discretization, e.g. implicit Runge–Kutta methods. During the last decades, efficient discontinuous Galerkin finite element methods have been presented for the time discretization of parabolic problems, see, e.g. an analysis for Galerkin time-stepping methods in [1–3], we also refer to the monograph [4]. Adaptive algorithms based on a posteriori error estimates have also been presented and successfully tested for linear and nonlinear problems, see e.g. [5,6] and the references therein. In [7,8], space-time adaptive wavelet methods for parabolic evolution problems have been studied. Also in the literature, *p* and *hp* finite element methods for parabolic problems have been presented, see [9,10].

Another approach that has been followed is the derivation of space-time finite element methods, based on appropriate space-time variational settings. The basic idea is to consider the time variable *t* as just another variable, lets say $x_{d+1}$, if we consider that $x=(x_{1},\ldots ,x_{d})$ are the spatial variables. In that way, the time derivative, which appears in the parabolic PDE model, plays the role of a convection term in the time direction $x_{d+1}$. By multiplying the given parabolic problem by a space-time test function and applying integration by parts, we can derive the weak space-time formulation. The derived weak formulation helps on the unified space-time discretization by finite element techniques. This means that we discretize the problem in space and in time by using a common finite element space. In this spirit, in [11], space-time finite element methods have been developed for elastodynamics. In particular, the method uses discontinuous Galerkin techniques for the time discretization and incorporates Petrov–Galerkin techniques, see [12], to ensure stability. Stream-line diffusion techniques that are presented in [12], have been also used for developing space-time finite element methods for conservation laws and fluid flow problems, see e.g. [13,14] and the references there. In [15,16], the stability of Petrov–Galerkin discretizations of parabolic problems have been studied and stable space-time trial and test functions have been proposed. In [17], conforming space-time finite element approximations to parabolic problems have been investigated. In [18], upwind-stabilized single-patch space-time Isogeometric analysis (IgA) schemes for parabolic evolution problems are proposed. Recently in [19,20], the authors based on [18], analyzed a time discontinuous Galerkin multipatch IgA scheme and demonstrated the efficiency of a space-time solver implemented on a parallel environment.

In this paper, we focus on the model problem $\partial_{t}u-\kappa \Delta u=f$, with zero initial and boundary conditions, and the diffusivity parameter *κ* is positive and constant. Following standard procedures, e.g. Nitsche techniques for imposing weakly boundary conditions, see [21], the current analysis can be extended to more general initial boundary problems. We propose a new space-time finite element method, which is stabilized by introducing classical bubble spaces, see [22–24]. The bubble basis functions vanish on the edges of the mesh elements and, in addition, do not affect the continuity properties of the solution. By enriching in that way the initial finite element space, the numerical solution consists of two components, where the first lives in the initial finite element space, and the second lives in the bubble space. For developing our analysis, we are motivated and inspired by the subgrid scale stabilization techniques presented in [25,26], for solving linear first-order problems. There, the idea is to couple the initial finite element space with new subgrid scale spaces and to construct artificial diffusion terms in these new spaces. The artificial diffusion terms are added in the numerical scheme in order to ensure stability. The innovation in our approach is that, instead of using subgrid spaces on different meshes, we use bubble spaces and the artificial diffusion terms are formed in these spaces. We include a positive parameter *θ* in the corresponding bubble diffusion terms, that can control the magnitude of the artificial diffusion in the time direction. We prove stability of the discrete problem with respect to the produced norm, which is a mesh depended norm. Also, optimal error estimates for the full numerical solution containing the bubble component are shown. The latter is achieved by choosing the value of *θ* to be close to the mesh size but independent of *κ*. During the discretization error analysis, we analytically present the dependence of the constants on to the diffusivity parameter *κ*. In the end, this helps to have a clear idea, about the form of the constants that appear in the error bounds for the difference between the solution *u* and the discrete solution $u_{h}$. In Section 5, we perform tests for different values of *κ*. To author's knowledge, it is the first time that this type of stabilized space-time finite element methods are presented and analyzed.

The paper is structured as follows. In Section 2, the model parabolic problem is presented. In Section 3, we formulate the stabilized space-time finite element scheme. In Section 4, we present the error analysis and derive the error estimates. We discuss numerical examples in Section 5. The paper closes with the conclusions.

## 2. The model problem

### 2.1. Preliminaries

Let Ω be a bounded Lipschitz domain in $R^{d},\;d\geq 1$. Let $\alpha =(\alpha_{1},\ldots ,\alpha_{d})$ be a multi-index of non-negative integers $\alpha_{1},\ldots ,\alpha_{d}$ with degree $|\alpha |=\sum_{j=1}^{d}\alpha_{j}$. For any α, we define the differential operator $D_{x}^{\alpha }=D_{x_{1}}^{\alpha_{1}}\ldots D_{x_{d}}^{\alpha_{d}}$, with $D_{x_{j}}=\partial /\partial x_{j}$, j=1,…,d. As usual, $L^{2}(\Omega )$ denotes the Sobolev space for which $\int_{\Omega }|\varphi (x){|}^{2}\;dx<\infty$, endowed with the norm $∥\varphi {∥}_{L^{2}(\Omega )}=(\int_{\Omega }|\varphi (x){|}^{2}\;dx{)}^{1/2}$. Let ℓ be a non-negative integer, define

$$H^{\ell }(\Omega )=\{\varphi \in L^{2}(\Omega ):D_{x}^{\alpha }\varphi \in L^{2}(\Omega ),\;\mathrm{for}\;\mathrm{all}\;|\alpha |\leq \ell \},$$

the standard Sobolev spaces endowed with the following norms $∥\varphi {∥}_{H^{\ell }(\Omega )}=(\sum_{0\leq |\alpha |\leq \ell }∥D_{x}^{\alpha }\varphi {∥}_{L^{2}(\Omega )}^{2}{)}^{1/2},$ and seminorms $|\varphi {|}_{H^{\ell }(\Omega )}=(\sum_{|\alpha |=\ell }∥D_{x}^{\alpha }\varphi {∥}_{L^{2}(\Omega )}^{2}{)}^{1/2}$. Also we define the subspace $H_{0}^{1}(\Omega )$ of $H^{1}(\Omega )$

$$H_{0}^{1}(\Omega )=\{\varphi \in H^{1}(\Omega ):\varphi =0\;\mathrm{on}\;\partial \Omega \}.$$

**Figure 1.**
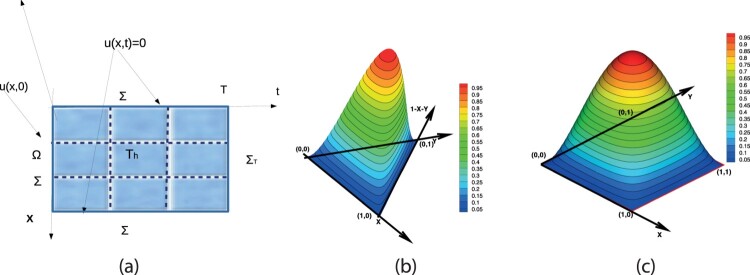
(a) The space-time domain *Q* with the boundary parts and its mesh $T_{h}(Q)$. (b) The bubble function on the reference triangular mesh element. (c) The bubble function on the reference rectangular mesh element.

Let I=[0,T] with *T*>0 be the time interval. For later use, we define the space-time cylinder Q=Ω×(0,T) and its boundary parts Σ=∂Ω×(0,T), $\Sigma_{T}=\Omega \times \{T\}$ and $\Sigma_{0}=\Omega \times \{0\},$ see an illustration Figure 1(a). We denote the gradient by $\nabla \varphi =(\nabla_{x}\varphi ,\partial_{t}\varphi )$, where $\nabla_{x}\varphi$ is the gradient with respect to the spatial variables. Similarly, we denote by $n=(n_{x},n_{t})$ the normal component on ∂Q, with $n_{x}$ the components related to space direction and $n_{t}$ the component related to time direction. Let ℓ,m be positive integers, for functions defined in *Q*, we define the Sobolev spaces

(1a) $$H^{\ell ,m}(Q)=\{\varphi \in L^{2}(Q):D_{x}^{\alpha }\varphi \in L^{2}(Q)\;\mathrm{with}\;0\leq |\alpha |\leq \ell ,\;\mathrm{and}\;\partial_{t}^{i}\varphi \in L^{2}(Q),\;i=1,\ldots ,m\}$$

and the subspaces

(1b) $$H_{0}^{1,0}(Q)=\{\varphi \in L^{2}(Q):\nabla_{x}\varphi \in [L^{2}(Q){]}^{d},\;\varphi =0\;\mathrm{on}\;\Sigma \},$$

(1c) $$H_{0,\bar{0}}^{1,1}(Q)=\{\varphi \in L^{2}(Q):\nabla_{x}\varphi \in [L^{2}(Q){]}^{d},\;\partial_{t}\varphi \in L^{2}(Q),\varphi =0\;\mathrm{on}\;\Sigma ,\varphi =0\;\mathrm{on}\;\Sigma_{T}\},$$

(1d) $$H_{0,\underline{0}}^{1,1}(Q)=\{\varphi \in L^{2}(Q):\nabla_{x}\varphi \in [L^{2}(Q){]}^{d},\;\partial_{t}\varphi \in L^{2}(Q),\varphi =0\;\mathrm{on}\;\Sigma ,\;\varphi =0\;\mathrm{on}\;\Sigma_{0}\}.$$

For a function $\varphi \in H^{\ell ,m}(Q)$ with ℓ,m≥1, we define the norms and the seminorms

(2a) $$∥\varphi {∥}_{H^{\ell ,m}(Q)}:={\left(\sum_{|\alpha |\leq \ell }∥D^{\alpha }\varphi {∥}_{L^{2}(Q)}^{2}+\sum_{i=0}^{m}∥\partial_{t}^{i}\varphi {∥}_{L^{2}(Q)}^{2}\right)}^{1/2},$$

(2b) $$|\varphi {|}_{H^{\ell ,m}(Q)}:={\left(\sum_{|\alpha |=\ell }∥D^{\alpha }\varphi {∥}_{L^{2}(Q)}^{2}+∥\partial_{t}^{m}\varphi {∥}_{L^{2}(Q)}^{2}\right)}^{1/2}.$$

We recall Hölder's and Young's inequalities

(3) $$\left|\int_{Q}uv\;dx\right|\leq ∥u{∥}_{L^{2}(Q)}∥v{∥}_{L^{2}(Q)}\;\text{and}\;\left|\int_{Q}uv\;dx\right|\leq \frac{\epsilon }{2}∥u{∥}_{L^{2}(Q)}^{2}+\frac{1}{2\epsilon }∥v{∥}_{L^{2}(Q)}^{2},$$

that hold for all $u\in L^{2}(Q)$ and $v\in L^{2}(Q)$ and for any fixed ϵ∈(0,∞).

We will use the following Poincare's inequality: Let $\Omega \subset R^{d},\;d=1,2,\ldots$ be a bounded rectangular domain and let Γ⊂∂Ω with |Γ|>0. For simplicity we assume that Γ lies on the plane with $x_{1}=0$. Let $v\in C^{\infty }(\Omega )$ and $v(x_{\Gamma })=0$ for all $x_{\Gamma }\in \Gamma$. For any interior point $x=(x_{1},\ldots ,x_{d})$, we have

(4) $$v(x_{1},\ldots ,x_{d})=v(x_{\Gamma })+\int_{x_{\Gamma ,1}}^{x_{1}}\frac{\partial v}{\partial x_{1}}(\tau ,x_{2},\ldots ,x_{d})\;d\tau .$$

The first inequality in (3) yields

(5) $$(v(x_{1},\ldots ,x_{d}){)}^{2}={\left(\int_{x_{\Gamma ,1}}^{x_{1}}1\;\frac{\partial v}{\partial x_{1}}(\tau ,x_{2},\ldots ,x_{d})\;d\tau \right)}^{2}\leq C_{\Omega }\int_{x_{\Gamma ,1}}^{x_{1}}{\left|\frac{\partial v}{\partial x_{1}}(\tau ,x_{2},\ldots ,x_{d})\right|}^{2}\;d\tau ,$$

where the constant $C_{\Omega }$ depends on the length of Ω. Integrating (5) over Ω, we can obtain

(6) $$\int_{\Omega }v^{2}(x)\;dx\leq C_{\Omega }^{2}\int_{\Omega }(\partial_{x_{1}}v{)}^{2}\;dx.$$

We refer to [27] for a proof of (6) for more general domains Ω.

In what follows, positive constants *c* and *C* appearing in inequalities are generic constants which do not depend on the mesh-size *h* and on *u*. In many cases, we will indicate on what may the constants depend for an easier understanding of the proofs. We will write a∼b meaning that ca≤b≤Ca, with c,C generic constants.

### 2.2. The model parabolic problem

In the space-time cylinder $\bar{Q}=\bar{\Omega }\times [0,T]$, with $\Omega \subset R^{d},\;d=1,2,3$, we consider the initial boundary value problem

(7) $$\begin{array}{rl} u_{t}-\kappa \Delta u & =f\;\mathrm{in}\;Q\;\text{and}\;\\ u & =0\;\mathrm{on}\;\Sigma ,\;u(\cdot ,0)=u_{0}\;\mathrm{on}\;\Sigma_{0}, \end{array}$$

as a model problem, where the diffusivity parameter 0<κ≤1 is taken to be constant, f:Q→R, with $f\in L^{2}(Q)$, and $u_{0}:\Omega \to R$, with $u_{0}\in L^{2}(\Omega )$ are given functions, and $u:\bar{Q}\to R$ is the unknown. Using the standard procedure and integration by parts with respect to both *x* and *t*, we can easily derive the following space-time variational formulation of (7): find $u\in H_{0}^{1,0}(Q)$ such that

(8) $$\bar{a}(u,v)=\bar{l}(v),\;\mathrm{for}\;\mathrm{all}\;v\in H_{0,\bar{0}}^{1,1}(Q)$$

with

(9) $$\bar{a}(u,v)=-\int_{Q}u(x,t)\;\partial_{t}v(x,t)\;dx\;dt+\kappa \int_{Q}\nabla_{x}u(x,t)\cdot \nabla_{x}v(x,t)\;dx\;dt,$$

(10) $$\bar{l}(v)=\int_{Q}f(x,t)v(x,t)\;dx\;dt+\int_{\Omega }u_{0}(x)v(x,0)\;dx.$$

The variational problem (8) is known to have a unique weak solution, see [28] and in [29,30] for a more comprehensive analysis on existence and uniqueness results. For simplicity, we only consider homogeneous Dirichlet boundary conditions on Σ and $u_{0}=0$. However, the analysis presented in this paper can easily be generalized to other constellations of boundary conditions.

Assumption 2.1 We assume that the solution *u* of (8) belongs to $V_{0,\underline{0}}:=H_{0,\underline{0}}^{1,1}(Q)\cap H^{s}(Q)$ with some s≥2.

Note that in Assumption 2.1 we require uniform regularity properties for the solution in *x* and *t* directions. In the following Sections we are going to present the discretization error analysis based on Assumption 2.1. In [19,20], error estimates have been given for Isogeometric Analysis methods considering anisotropic regularity properties for the solution *u*.

## 3. The discrete problem

### 3.1. A usual finite element scheme

Let $T_{h}(Q)$ be a partition of space-time domain *Q* into triangular (or quadrilateral elements), that is $\bar{Q}=\cup_{E\in T_{h}}E$, see Figure 1(a). We denote by $h_{E}$ the diameter of $E\in T_{h}(Q)$ and the mesh size is defined as $h=\max_{E}\{h_{E}\}$. We assume that $T_{h}(Q)$ is uniform, i.e. there exists a positive number $C_{un}$, such that $\rho_{E}\geq h/C_{un}$, where $\rho_{E}$ is the diameter of the circle inscribed in $E\in T_{h}(Q)$. Associated with $T_{h}(Q)$, we define the finite element subspace $V_{h0}$ of $H_{0,\underline{0}}^{1,1}(Q)$, consisting of continuous functions in space and in time, by

(11) $$V_{h0}=\{v_{h}\in H_{0,\underline{0}}^{1,1}(Q):v_{h}{|}_{E}\in P^{p}(E),\;\mathrm{for}\;\mathrm{every}\;E\in T_{h}(Q)\},$$

where $P^{p}(E)$ is the polynomial space of total degree *p*, see e.g. [31–33]. In our analysis we focus on the case *p*=1.

The usual finite element approximation of (7) would read: find $u_{h}\in V_{h0}$ such that

(12a) $$a(u_{h},v_{h})=(f,v_{h})\;\forall \;v_{h}\in V_{h0},$$

where

(12b) $$a(u_{h},v_{h})=\int_{Q}\partial_{t}u_{h}\;v_{h}\;dx\;dt+\kappa \int_{Q}\nabla_{x}u_{h}\cdot \nabla_{x}v_{h}\;dx\;dt.$$

Setting $v_{h}=u_{h}$ in (12a) and using the identity $2\int_{Q}\partial u_{h}u_{h}\;dx\;dt=\int_{Q}\partial_{t}(u_{h}^{2})\;dx\;dt=\int_{\Sigma_{T}}u_{h}^{2}\;ds$, we have $∥u_{h}{∥}_{L^{2}(\Sigma_{T})}^{2}+\kappa ∥\nabla_{x}u_{h}{∥}_{L^{2}(Q)}^{2}\leq ∥f{∥}_{L^{2}(Q)}∥u_{h}{∥}_{L^{2}(Q)}$, and by inequalities (3) and (6), we can obtain the following estimate

(13) $$\frac{1}{2}∥u_{h}{∥}_{L^{2}(\Sigma_{T})}^{2}+\frac{\kappa }{2}∥\nabla_{x}u_{h}{∥}_{L^{2}(Q)}^{2}\leq \frac{2C_{\Omega }^{2}}{\kappa }∥f{∥}_{L^{2}(Q)}^{2},$$

where $C_{\Omega }$ is the constant that appears in (6). In the stability estimate (13) the control of $\nabla_{x}u_{h}$ can be very poor if the parameter *κ* is very small. Furthermore (13) does not provide a direct control of $∥\partial_{t}u_{h}{∥}_{L^{2}(Q)}$. Thus in the case where *κ* is small, the finite element scheme (12) may not perform well. Therefore it is crucial to improve the stability properties of (12). Next we present a technique for stabilizing the finite element scheme (12) by adding artificial diffusion terms, which do not deteriorate the approximate properties of the method. The idea is to enrich the original finite dimensional space by adding a bubble function space, and then to construct the artificial diffusion terms in this space.

### 3.2. The stabilized scheme

We introduce the larger finite subspace $V_{h,b}$ of $H_{0,\underline{0}}^{1,1}(Q)$ that can be written as a direct sum as follows

(14) $$V_{h,b}=\{v_{h}\in H_{0,\underline{0}}^{1,1}(Q):v_{h}{|}_{E}\in P^{1}(E)⊕V_{B}(E),\;\mathrm{for}\;\mathrm{every}\;E\in T_{h}(Q)\},$$

with $V_{B}(E):=V_{B}{|}_{E}$, where $V_{B}$ denotes the space of bubble functions, that vanish entirely on the boundary of the mesh elements and have exactly one degree of freedom in each $E\in T_{h}(Q)$. For example, in case of triangular elements, it is spanned by a cubic functions $V_{B}:=\{v^{b}\in H_{0}^{1}(Q):v^{b}{|}_{E}=C_{E}\lambda_{1}\lambda_{2}\lambda_{3}\}$, where $\lambda_{i},i=1,2,3$ are linear polynomials vanishing on one side of ∂E and taking the value one at the opposite vertex. The constant $C_{E}$ is chosen such that $\max_{x\in E}v^{b}(x)=1$. Thus, every bubble basis function $\varphi \in V_{B}(E)$ satisfies, (i) φ(x)>0 for x∈E, (ii) φ(x)=0 for x∈∂E, and (iii) $\int_{E}\varphi^{2}(x)\;dx=C_{E}h_{E}^{2}$, with $C_{E}$ depending on the uniformity of $T_{h}$ but is independent of $h_{E}$. An illustration of bubble functions on two-dimensional elements is presented in Figure 1(b,c).

Based on $V_{h,b}$ defined in (14), any $v_{h}\in V_{h,b}$ can be decomposed into two parts, i.e. $v_{h}=v_{h}^{1}+v_{h}^{b}$ with $v_{h}^{1}\in V_{h0}$ and $v_{h}^{b}\in V_{B}$. In view of this, we introduce the discrete problem: find $u_{h}\in V_{h,b}$ such that

(15a) $$a(u_{h},v_{h})+b_{h}(u_{h}^{b},v_{h}^{b})=(f,v_{h}),\;\forall \;v_{h}\in V_{h,b},$$

where a(⋅,⋅) as in (12b) and

(15b) $$b_{h}(u_{h}^{b},v_{h}^{b})=\theta h\int_{Q}\partial_{t}u_{h}^{b}\;\partial_{t}v_{h}^{b}\;dx\;dt,$$

with θ>0 to be a positive constant, which will be determined later. We recall the following inverse estimate and the scaled trace inequality, see proofs in [31] and in [21].

Lemma 3.1. Let $v\in H^{1}(Q),\;v_{h}\in V_{h,b}$ and let a mesh element $E\in T_{h}(Q)$. Then there exist constants $c_{\mathrm{inv}},\;c_{\mathrm{trac}}>0$ independent of *h* such that

(16) $$∥\nabla v_{h}{∥}_{L^{2}(E)}\leq c_{\mathrm{inv}}h^{-1}∥v_{h}{∥}_{L^{2}(E)},$$

(17) $$∥v{∥}_{L^{2}(\partial E)}\leq c_{\mathrm{trac}}h^{-1/2}(∥v{∥}_{L^{2}(E)}+h∥\nabla v{∥}_{L^{2}(E)}).$$

For convenience, we introduce the discrete bilinear form

(18) $$a_{h}(u_{h},v_{h})=a(u_{h},v_{h})+b_{h}(u_{h}^{b},v_{h}^{b}),$$

and the associated mesh dependent norm

(19) $$∥v_{h}{∥}_{h}=(\kappa ∥\nabla_{x}v_{h}{∥}_{L^{2}(Q)}^{2}+\theta h∥\partial_{t}v_{h}^{b}{∥}_{L^{2}(Q)}^{2}+\frac{1}{2}∥v_{h}{∥}_{L^{2}(\Sigma_{T})}^{2}{)}^{1/2}.$$

Note that, the terms with the time derivatives in (19) are related to the bubble function space.

Lemma 3.2. The discrete form $a_{h}(\cdot ,\cdot ):V_{h,b}\times V_{h,b}\to R$ defined in (18), is $V_{h,b}$-coercive with respect to the norm $∥\cdot {∥}_{h},$ i.e.,

(20) $$a_{h}(v_{h},v_{h})\geq C_{s}∥v_{h}{∥}_{h}^{2},\;\forall \;v_{h}\in V_{h,b}.$$

Proof. Let $v_{h}\in V_{h,b}$. Since $v_{h}(x,0)=0$ and $n_{t}{|}_{\Sigma }=0$, it follows by Green's formula $\int_{Q}\partial_{t}v_{h}v_{h}+v_{h}\partial_{t}v_{h}\;dx\;dt=\int_{\partial Q}n_{t}v_{h}^{2}\;ds,$ that

(21) $$\int_{Q}\partial_{t}v_{h}\;v_{h}\;dx\;dt=\frac{1}{2}\int_{Q}\partial_{t}v_{h}^{2}\;dx\;dt=\frac{1}{2}\int_{\Sigma_{T}}v_{h}^{2}\;ds-\frac{1}{2}\int_{\Sigma_{0}}v_{h}^{2}\;ds=\frac{1}{2}∥v_{h}{∥}_{L^{2}(\Sigma_{T})}^{2}.$$

The definition of $a_{h}$ and (21) imply

(22) $$\begin{array}{rl} a_{h}(v_{h},v_{h}) & =\int_{Q}\frac{1}{2}\partial_{t}v_{h}^{2}+\theta h(\partial_{t}v_{h}^{b}{)}^{2}+\kappa |\nabla_{x}v_{h}{|}^{2}\;dx\;dt\\ & =\frac{1}{2}∥v_{h}{∥}_{L^{2}(\Sigma_{T})}^{2}+\kappa ∥\nabla_{x}v_{h}{∥}_{L^{2}(Q)}^{2}+\theta h∥\partial_{t}v_{h}^{b}{∥}_{L^{2}(Q)}^{2}, \end{array}$$

which is (20) with $C_{s}=1$ and this completes the proof.

Proposition 3.1. Let $u_{h}$ be the solution given by (15). Then there is a $C_{\kappa ,\Omega }>0$ such that the solution $u_{h}$ satisfies the following a priori estimate

(23) $$∥u_{h}{∥}_{h}\leq C_{\kappa ,\Omega }∥f{∥}_{L^{2}(Q)}.$$

Proof. Using $u_{h}\in V_{h,b}$ as a test function in (15), and utilizing (3) and (20)) together with the Poincare inequality (6), we successively obtain

(24) $$\begin{array}{rl} C_{s}∥u_{h}{∥}_{h}^{2} & \leq a_{h}(u_{h},u_{h})\leq \frac{1}{\kappa^{1/2}}\left|\int_{Q}\kappa^{1/2}fu_{h}\;dx\;dt\right|\leq C_{\Omega }\frac{1}{\kappa^{1/2}}∥f{∥}_{L^{2}(Q)}∥\kappa^{1/2}\nabla_{x}u_{h}{∥}_{L^{2}(Q)}\\ & \leq C_{\Omega }\frac{1}{\kappa }∥f{∥}_{L^{2}(Q)}(\kappa ∥\nabla_{x}u_{h}{∥}_{L^{2}(Q)}^{2}+\frac{1}{2}∥u_{h}{∥}_{L^{2}(\Sigma_{T})}^{2}+\theta h∥\partial_{t}u_{h}^{b}{∥}_{L^{2}(Q)}^{2}{)}^{1/2}, \end{array}$$

where we have previously used that $C_{s}=1$ and κ≤1. Setting $C_{\kappa ,\Omega }=C_{\Omega }(1/\kappa )$ we get (23).

Note that the estimate in (23) provides a direct control of $∥\partial_{t}u_{h}^{b}{∥}_{L^{2}(Q)}^{2}$ due to the appearance of b(⋅,⋅) in the finite element scheme (15), cf. (13). A direct result of (23) and (20) is the following corollary.

Corollary 3.1. The discrete problem defined in (15) is well posed, i.e. it has a unique solution which satisfies the stability estimate (23).

Next, we show the boundedness of a(⋅,⋅) on $V_{0,\underline{0}}\times V_{h,b}$, where the space $V_{0,\underline{0}}$ is defined in Assumption 2.1. We define the norms

(25a) $$∥v{∥}_{h,*}=(\kappa ∥\nabla_{x}v{∥}_{L^{2}(Q)}^{2}+\theta h∥\partial_{t}v{∥}_{L^{2}(Q)}^{2}+\frac{1}{2}∥v{∥}_{L^{2}(\Sigma_{T})}^{2}{)}^{1/2},$$

(25b) $$∥v{∥}_{h,V}=(\kappa ∥\nabla_{x}v{∥}_{L^{2}(Q)}^{2}+\theta h∥\partial_{t}v{∥}_{L^{2}(Q)}^{2}+\frac{1}{2}∥v{∥}_{L^{2}(\Sigma_{T})}^{2}+(\theta h{)}^{-1}∥v{∥}_{L^{2}(Q)}^{2}{)}^{1/2}.$$

We point out that the $∥\cdot {∥}_{h,*}$ is defined for all $v\in V_{0,\underline{0}}$ and is similar to the $∥\cdot {∥}_{h}$ given in (19), which is defined for all $v_{h}\in V_{h,b}$. The mesh-dependent norm $∥\cdot {∥}_{h,V}$ will be used later for deriving bounds for the discretization error.

Lemma 3.3. There is a constant $C_{b}(\kappa ,\theta ,h)>0$ such that

(26) $$|a(u,v_{h})|\leq C_{b}(\kappa ,\theta ,h)∥u{∥}_{h,V}∥v_{h}{∥}_{h},\;\forall \;(u,v_{h})\in (V_{0,\underline{0}}\times V_{h,b}).$$

Proof. Let $v_{h}=v_{h}^{1}+v_{h}^{b}\in V_{h,b}$. We treat every term of the form a(⋅,⋅) separately. We apply integration by parts and (3) to infer

(27) $$\begin{array}{rl} \int_{Q}\partial_{t}uv_{h}\;dx\;dt & =-\int_{Q}u\partial_{t}v_{h}\;dx\;dt+\int_{\Sigma_{T}}uv_{h}\;d\sigma \\ & \leq ((\theta h{)}^{-1}∥u{∥}_{L^{2}(Q)}^{2}{)}^{1/2}((\theta h)∥\partial_{t}v_{h}{∥}_{L^{2}(Q)}^{2}{)}^{1/2}+∥u{∥}_{L^{2}(\Sigma_{T})}∥v_{h}{∥}_{L^{2}(\Sigma_{T})}\\ & \overset{\left(16\right)}{\leq }((\theta h{)}^{-1}∥u{∥}_{L^{2}(Q)}^{2}{)}^{1/2}{\left(\frac{c_{1}\theta h}{h^{2}\kappa }\kappa ∥v_{h}{∥}_{L^{2}(Q)}^{2}\right)}^{1/2}+2{\left(\frac{1}{2}∥u{∥}_{L^{2}(\Sigma_{T})}^{2}\right)}^{1/2}{\left(\frac{1}{2}∥v_{h}{∥}_{L^{2}(\Sigma_{T})}^{2}\right)}^{1/2}\\ & \overset{\left(6\right)}{\leq }{\left((\theta h{)}^{-1}∥u{∥}_{L^{2}(Q)}^{2}\right)}^{1/2}(c_{2}\theta (\kappa h{)}^{-1}{)}^{1/2}{\left(\kappa ∥\nabla_{x}v_{h}{∥}_{L^{2}(Q)}^{2}+\theta h∥\partial_{t}v_{h}^{b}{∥}_{L^{2}(Q)}^{2}+\frac{1}{2}∥v_{h}{∥}_{L^{2}(\Sigma_{T})}^{2}\right)}^{1/2}\\ & \;+2∥u{∥}_{L^{2}(\Sigma_{T})}{\left(\kappa ∥\nabla_{x}v_{h}{∥}_{L^{2}(Q)}^{2}+\theta h∥\partial_{t}v_{h}^{b}{∥}_{L^{2}(Q)}^{2}+\frac{1}{2}∥v_{h}{∥}_{L^{2}(\Sigma_{T})}^{2}\right)}^{1/2}\\ & \leq (c_{2}\theta (\kappa h{)}^{-1}{)}^{1/2}∥u{∥}_{h,V}∥v_{h}{∥}_{h}+2∥u{∥}_{h,V}∥v_{h}{∥}_{h}\leq c_{3}(\theta (\kappa h{)}^{-1}+1{)}^{1/2}∥u{∥}_{h,V}∥v_{h}{∥}_{h}, \end{array}$$

where $c_{3}$ depends on the constants appearing in (16) and (6). Similarly for the second term, applying (3), we get

(28) $$\int_{Q}\kappa^{1/2}\nabla_{x}u\cdot \kappa^{1/2}\nabla_{x}v_{h}\;dx\;dt\leq (\kappa ∥\nabla_{x}u{∥}_{L^{2}(Q)}^{2}{)}^{1/2}(\kappa ∥\nabla_{x}v_{h}{∥}_{L^{2}(Q)}^{2}{)}^{1/2}\leq ∥u{∥}_{h,V}∥v_{h}{∥}_{h}.$$

Combining all the above bounds and setting $C_{b}(\kappa ,\theta ,h)=2c_{3}(\theta (\kappa h{)}^{-1}+1{)}^{1/2}$, we can derive the desired result.

## 4. Error analysis

Proposition 4.1 **weak consistency**

Let Assumption 2.1 hold and let $u_{h}$ the solution given by (15), and furthermore let $z_{h}\in V_{h,b}$ and $v_{h}^{1}\in V_{h0}$. Then

(29a) $$a_{h}(u_{h},z_{h})=a(u,z_{h}),\;\mathrm{and}$$

(29b) $$a_{h}(v_{h}^{1},z_{h})=a(v_{h}^{1},z_{h}).$$

Proof. Multiplying (7) by $z_{h}\in V_{h,b}$, integrating over *Q*, and then applying integration by parts, we arrive at the variational identity

(30) $$a(u,z_{h})=(f,z_{h}).$$

We recall the problem (15) and directly have

(31) $$a_{h}(u_{h},z_{h})=(f,z_{h})=a(u,z_{h}).$$

Furthermore, we observe that $b_{h}(v_{h}^{1},z_{h})=0$ for any $v_{h}^{1}\in V_{h0}$, and (29b) directly follows.

Lemma 4.1. Let $u_{h}$ solve (15) and let $z_{h}^{1}\in V_{h0}$. Under Assumption 2.1, there exists a *c*, independent of *h* such that

(32) $$(∥u-u_{h}{∥}_{L^{2}(\Sigma_{T})}^{2}\;+\;\kappa ∥\nabla_{x}u-\nabla_{x}u_{h}{∥}_{L^{2}(Q)}^{2}\;+\;\theta h∥\partial_{t}u_{h}^{b}{∥}_{L^{2}(Q)}^{2})\;\leq \;\frac{c}{\kappa }(∥\nabla u-\nabla z_{h}^{1}{∥}_{L^{2}(Q)}^{2}\;+\;∥u-z_{h}^{1}{∥}_{L^{2}(\Sigma_{T})}^{2}).$$

Proof. Let $z_{h}^{1}$ be a function in $V_{h0}$. By (29) and by subtracting similar terms, we have that

(33) $$\begin{array}{rl} & \int_{Q}(\partial_{t}u_{h}-\partial_{t}z_{h}^{1})\varphi_{h}+\kappa \int_{Q}\nabla_{x}(u_{h}-z_{h}^{1})\cdot \nabla_{x}\varphi_{h}+\theta h\int_{Q}\partial_{t}u_{h}^{b}\partial_{t}\varphi_{h}^{b}\\ & \;=\int_{Q}(\partial_{t}u-\partial_{t}z_{h}^{1})\varphi_{h}+\kappa \int_{Q}\nabla_{x}(u-z_{h}^{1})\cdot \nabla_{x}\varphi_{h},\;\forall \;\varphi_{h}\in V_{h,b}. \end{array}$$

Setting above $\varphi_{h}=u_{h}^{1}+u_{h}^{b}-z_{h}^{1}$ and applying integration by parts on the first term on the left side, we have

(34) $$\begin{array}{rl} & \int_{\Sigma_{T}}|\partial_{t}u_{h}-\partial_{t}z_{h}^{1}{|}^{2}\;d\sigma +\kappa \int_{Q}|\nabla_{x}(u_{h}-z_{h}^{1}){|}^{2}\;dx\;dt+\theta h\int_{Q}|\partial_{t}u_{h}^{b}{|}^{2}\;dx\;dt\\ & \;\leq {\left(\frac{1}{\kappa }\int_{Q}|\partial_{t}u-\partial_{t}z_{h}^{1}{|}^{2}\right)}^{1/2}{\left(\kappa \int_{Q}|u_{h}-z_{h}^{1}{|}^{2}\right)}^{1/2}\\ & \;+{\left(\int_{Q}\kappa |\nabla_{x}(u-z_{h}^{1}){|}^{2}\right)}^{1/2}{\left(\int_{Q}\kappa |\nabla_{x}(u_{h}-z_{h}^{1}){|}^{2}\right)}^{1/2}, \end{array}$$

and by applying (3) and (4) on the right hand side, yields

(35) $$\begin{array}{rl} & ∥u_{h}-z_{h}^{1}{∥}_{L^{2}(\Sigma_{T})}^{2}+\kappa ∥\nabla_{x}u_{h}-\nabla_{x}z_{h}^{1}{∥}_{L^{2}(Q)}^{2}+\theta h∥\partial_{t}u_{h}^{b}{∥}_{L^{2}(Q)}^{2}\leq \frac{1}{c_{\epsilon }\kappa }∥\partial_{t}u-\partial_{t}z_{h}^{1}{∥}_{L^{2}(Q)}^{2}\\ & \;+c_{\epsilon }\kappa ∥\nabla_{x}u_{h}-\nabla_{x}z_{h}^{1}{∥}_{L^{2}(Q)}^{2}+\frac{\kappa }{c_{\epsilon }}∥\nabla_{x}u-\nabla_{x}z_{h}^{1}{∥}_{L^{2}(Q)}^{2}+c_{\epsilon }\kappa ∥\nabla_{x}u_{h}-\nabla_{x}z_{h}^{1}{∥}_{L^{2}(Q)}^{2}. \end{array}$$

Gathering the same terms and setting $0<c_{\epsilon }<\frac{1}{2}$, we get

(36) $$\begin{array}{rl} & \left(1-2c_{\epsilon })(∥u_{h}-z_{h}^{1}{∥}_{L^{2}(\Sigma_{T})}^{2}+\kappa ∥\nabla_{x}u_{h}-\nabla_{x}z_{h}^{1}{∥}_{L^{2}(Q)}^{2}+\theta h∥\partial_{t}u_{h}^{b}{∥}_{L^{2}(Q)}^{2}\right)\\ & \;\leq \frac{1}{c_{\epsilon }\kappa }∥\partial_{t}u-\partial_{t}z_{h}^{1}{∥}_{L^{2}(Q)}^{2}+\frac{\kappa }{c_{\epsilon }}∥\nabla_{x}u-\nabla_{x}z_{h}^{1}{∥}_{L^{2}(Q)}^{2}+∥u-z_{h}^{1}{∥}_{L^{2}(\Sigma_{T})}^{2}. \end{array}$$

Using 0<κ≤1, applying triangle inequality and setting $c=(1/\left(1-2\;c_{\epsilon }\right))(1/c_{\epsilon })$, the assertion follows.

Below we give the main error bound for the finite element solution $u_{h}\in V_{h,b}$.

Theorem 4.1. Let $u_{h}=u_{h}^{1}+u_{h}^{b}$ solve (15). Under Assumption 2.1 and choosing θ≥h, there exist a constant $c_{*,V},$ depending on $c_{\mathrm{inv}}$ in (16), such that

(37) $$∥u-u_{h}{∥}_{h,*}^{2}\leq c_{*,V}^{2}(\mu_{1}(\kappa ,\theta ,h)∥u-z_{h}^{1}{∥}_{h,V}^{2}+\mu_{2}(\kappa ,\theta ,h)∥u-z_{h}^{1}{∥}_{L^{2}(Q)}^{2}),\;for\;z_{h}^{1}\in V_{h0},$$

where $\mu_{1}(\kappa ,\theta ,h)=(1+{\tilde{\gamma }}^{2}(\kappa ,\theta ,h)+{\tilde{\gamma }}^{3}(\kappa ,\theta ,h)+\tilde{\gamma }(\kappa ,\theta ,h))$ and $\mu_{2}(\kappa ,\theta ,h)={\tilde{\gamma }}^{2}(\kappa ,\theta ,h)h^{-2}$, with $\tilde{\gamma }(\kappa ,\theta ,h)=(\theta h{)}^{1/2}\gamma (\kappa ,\theta ,h)$ and $\gamma (\kappa ,\theta ,h)=(1/(\theta \;h{)}^{1/2}+c_{\mathrm{inv}}\kappa^{1/2}/h)$.

Proof. Let us consider the functions $z_{h}^{1}\in V_{h0}$ and $\sigma_{h}=(u_{h}^{1}+u_{h}^{b})-z_{h}^{1}$. Using triangle inequality, we decompose the error as

(38) $$\begin{array}{rl} & \frac{1}{2}∥u-u_{h}{∥}_{h,*}^{2}=\frac{1}{2}((\theta h)∥\partial_{t}u-\partial_{t}u_{h}{∥}_{L^{2}(Q)}^{2}+\kappa ∥\nabla_{x}u-\nabla_{x}u_{h}{∥}_{L^{2}(Q)}^{2}+\frac{1}{2}∥u-u_{h}{∥}_{L^{2}(\Sigma_{T})}^{2})\\ & \;\leq \overset{T_{1}}{\overbrace{(\theta h)∥\partial_{t}u-\partial_{t}z_{h}^{1}{∥}_{L^{2}(Q)}^{2}+\kappa ∥\nabla_{x}u-\nabla_{x}z_{h}^{1}{∥}_{L^{2}(Q)}^{2}+\frac{1}{2}∥u-z_{h}^{1}{∥}_{L^{2}(\Sigma_{T})}^{2}}}+\overset{T_{2}}{\overbrace{(\theta h)∥\partial_{t}u_{h}^{1}-\partial_{t}z_{h}^{1}{∥}_{L^{2}(Q)}^{2}}}\\ & \;+\overset{T_{3}}{\overbrace{(\theta h)∥\partial_{t}u_{h}^{b}-0\cdot \partial_{t}z_{h}^{b}{∥}_{L^{2}(Q)}^{2}+\kappa ∥\nabla_{x}u_{h}-\nabla_{x}z_{h}^{1}{∥}_{L^{2}(Q)}^{2}+\frac{1}{2}∥u_{h}-z_{h}^{1}{∥}_{L^{2}(\Sigma_{T})}^{2}}}\\ & \;\leq ∥u-z_{h}^{1}{∥}_{h,V}^{2}+T_{2}+T_{3}, \end{array}$$

where we previously set $T_{1}:=∥u-z_{h}^{1}{∥}_{h,*}^{2}$, $T_{2}:=(\theta h)∥\partial_{t}u_{h}^{1}-\partial_{t}z_{h}^{1}{∥}_{L^{2}(Q)}^{2}$, $T_{3}:=∥\sigma_{h}{∥}_{h}^{2}$, and in the last inequality we used that $T_{1}\leq ∥u-z_{h}^{1}{∥}_{h,V}^{2}.$ We will proceed by giving bounds for every term appearing in (38). We first show few auxiliary results. Let $v_{h}\in V_{h,b}$ and $\sigma_{h}^{1}=u_{h}^{1}-z_{h}^{1}$, then using (16), (18) and (29) and the fact that 0<h,θ,κ≤1, we can obtain that

(39) $$\begin{array}{rl} & ∥\partial_{t}u_{h}^{1}-\partial_{t}z_{h}^{1}{∥}_{L^{2}(Q)}=∥\partial_{t}\sigma_{h}^{1}{∥}_{L^{2}(Q)}\leq \underset{v_{h}\in V_{h,b}}{\sup}\frac{|\int_{Q}\partial_{t}((u_{h}^{1}+u_{h}^{b})-z_{h}^{1}-u_{h}^{b})v_{h}\;dx\;dt|}{∥v_{h}{∥}_{L^{2}(Q)}}\\ & \;\leq \underset{v_{h}\in V_{h,b}}{\sup}\frac{\left|-\left(\int_{Q}\partial_{t}u_{h}^{b}v_{h}+\kappa \nabla_{x}\sigma_{h}\cdot \nabla_{x}v_{h}+\theta h\partial_{t}u_{h}^{b}\partial_{t}v_{h}^{b}\;dx\;dt\right)\right|}{∥v_{h}{∥}_{L^{2}(Q)}}\\ & \;+\underset{v_{h}\in V_{h,b}}{\sup}\frac{\left|\int_{Q}\partial_{t}(u-\partial_{t}z_{h}^{1})v_{h}+\kappa \nabla_{x}(u-z_{h}^{1})\cdot \nabla_{x}v_{h}\right|}{∥v_{h}{∥}_{L^{2}(Q)}}\\ & \;\overset{\left(16\right)}{\leq }\frac{(\theta h{)}^{1/2}}{(\theta h{)}^{1/2}}(∥\partial_{t}u_{h}^{b}{∥}_{L^{2}(Q)}+c_{\mathrm{inv}}\theta ∥\partial_{t}u_{h}^{b}{∥}_{L^{2}(Q)}))+∥\partial_{t}(u-z_{h}^{1}){∥}_{L^{2}(Q)}\\ & \;+\kappa^{1/2}(\kappa^{1/2}∥\nabla_{x}(u-z_{h}^{1}){∥}_{L^{2}(Q)}+\kappa^{1/2}∥\nabla_{x}\sigma_{h}{∥}_{L^{2}(Q)}))\underset{v_{h}\in V_{h,b}}{\sup}\frac{∥\nabla v_{h}{∥}_{L^{2}(Q)}}{∥v_{h}{∥}_{L^{2}(Q)}}\\ & \;\leq \frac{c_{0}}{(\theta h{)}^{1/2}}(\theta h∥\partial_{t}u_{h}^{b}{∥}_{L^{2}(Q)}^{2}+\kappa ∥\nabla_{x}\sigma_{h}{∥}_{L^{2}(Q)}^{2}+\frac{1}{2}∥\sigma_{h}{∥}_{L^{2}(\Sigma_{T})}^{2}{)}^{1/2}\\ & \;+\frac{c_{\mathrm{inv}}\kappa^{1/2}}{h}(\theta h∥\partial_{t}u_{h}^{b}{∥}_{L^{2}(Q)}^{2}+\kappa ∥\nabla_{x}\sigma_{h}{∥}_{L^{2}(Q)}^{2}+\frac{1}{2}∥\sigma_{h}{∥}_{L^{2}(\Sigma_{T})}^{2}{)}^{1/2}\\ & \;+\frac{c_{\mathrm{inv}}\kappa^{1/2}}{h}(\kappa^{1/2}∥\nabla_{x}(u-z_{h}^{1}){∥}_{L^{2}(Q)})+\frac{(\theta h{)}^{1/2}}{(\theta h{)}^{1/2}}∥\partial_{t}(u-z_{h}^{1}){∥}_{L^{2}(Q)}\\ & \;\leq c_{0}\left(\frac{1}{(\theta h{)}^{1/2}}+\frac{c_{\mathrm{inv}}\kappa^{1/2}}{h}\right)∥\sigma_{h}{∥}_{h}+\left(\frac{1}{(\theta h{)}^{1/2}}+\frac{c_{\mathrm{inv}}\kappa^{1/2}}{h}\right)∥u-z_{h}^{1}{∥}_{h,V}\\ & \;\leq c_{0}\gamma (\kappa ,\theta ,h)(∥u-z_{h}^{1}{∥}_{h,V}+∥\sigma_{h}{∥}_{h}), \end{array}$$

where we previously set $c_{0}=(1+\theta c_{\mathrm{inv}})$. Using (39) and the relation

(40) $$(\theta h{)}^{1/2}∥\partial_{t}u_{h}^{1}-\partial_{t}z_{h}^{1}{∥}_{L^{2}(Q)}+(\theta h{)}^{1/2}∥\partial_{t}u_{h}^{b}-0\partial_{t}z_{h}^{b}{∥}_{L^{2}(Q)}\geq (\theta h{)}^{1/2}∥\partial_{t}(u_{h}^{1}+u_{h}^{b})-\partial_{t}z_{h}^{1}{∥}_{L^{2}(Q)},$$

and observing that $(\theta h{)}^{1/2}\gamma (\kappa ,\theta ,h)\geq 1$, it follows that

(41) $$(\theta h{)}^{1/2}∥\partial_{t}\sigma_{h}{∥}_{L^{2}(Q)}\leq 2c_{0}(\theta h{)}^{1/2}\gamma (\kappa ,\theta ,h)(∥u-z_{h}^{1}{∥}_{h,V}+∥\sigma_{h}{∥}_{h}).$$

Furthermore, working as in the proof of (26) and using (41) and (3), we can find

(42) $$\begin{array}{rl} & a(u-z_{h}^{1},\sigma_{h})\\ & \;=-\int_{Q}(u-z_{h}^{1})\partial_{t}\sigma_{h}\;dx\;dt+\int_{\Sigma_{T}}(u-z_{h}^{1})\sigma_{h}d\sigma +\int_{Q}\kappa^{1/2}\nabla_{x}(u-z_{h}^{1})\cdot \kappa^{1/2}\nabla_{x}\sigma_{h}\;dx\;dt\\ & \;\leq ((\theta h{)}^{-1}∥u-z_{h}^{1}{∥}_{L^{2}(Q)}^{2}{)}^{1/2}((\theta h)∥\partial_{t}\sigma_{h}{∥}_{L^{2}(Q)}^{2}{)}^{1/2}+∥u-z_{h}^{1}{∥}_{L^{2}(\Sigma_{T})}∥\sigma_{h}{∥}_{L^{2}(\Sigma_{T})}\\ & \;+(\kappa ∥\nabla_{x}(u-z_{h}^{1}){∥}_{L^{2}(Q)}^{2}{)}^{1/2}(\kappa ∥\nabla_{x}\sigma_{h}{∥}_{L^{2}(Q)}^{2}{)}^{1/2}\\ & \;\leq 2c_{0}((\theta h{)}^{-1}∥u-z_{h}^{1}{∥}_{L^{2}(Q)}^{2}{)}^{1/2}(\theta h{)}^{1/2}\gamma (\kappa ,\theta ,h)(∥u-z_{h}^{1}{∥}_{h,V}+∥\sigma_{h}{∥}_{h})+2∥u-z_{h}^{1}{∥}_{h,V}∥\sigma_{h}{∥}_{h}\\ & \;\leq (\theta h{)}^{1/2}\gamma (\kappa ,\theta ,h)∥u-z_{h}^{1}{∥}_{h,V}^{2}+2c_{0}h^{-1}∥u-z_{h}^{1}{∥}_{L^{2}(Q)}h\gamma (\kappa ,\theta ,h)∥\sigma_{h}{∥}_{h}+2∥u-z_{h}^{1}{∥}_{h,V}∥\sigma_{h}{∥}_{h}\\ & \;\leq (\theta h{)}^{1/2}\gamma (\kappa ,\theta ,h)∥u-z_{h}^{1}{∥}_{h,V}^{2}+2c_{\varepsilon }h^{-2}∥u-z_{h}^{1}{∥}_{L^{2}(Q)}^{2}+\varepsilon h^{2}\gamma^{2}(\kappa ,\theta ,h)∥\sigma_{h}{∥}_{h}^{2}\\ & \;+c_{\varepsilon }∥u-z_{h}^{1}{∥}_{h,V}^{2}+\varepsilon ∥\sigma_{h}{∥}_{h}^{2}\\ & \;\leq (\theta h{)}^{1/2}\gamma (\kappa ,\theta ,h)∥u-z_{h}^{1}{∥}_{h,V}^{2}+2c_{\varepsilon }h^{-2}∥u-z_{h}^{1}{∥}_{L^{2}(Q)}^{2}+\varepsilon (h\theta^{-1}+c_{\mathrm{inv}}\kappa )∥\sigma_{h}{∥}_{h}^{2}\\ & \;+c_{\varepsilon }∥u-z_{h}^{1}{∥}_{h,V}^{2}+\varepsilon ∥\sigma_{h}{∥}_{h}^{2},\;\text{(using that }\theta \geq h\text{)}\\ & \;\leq 2c_{\varepsilon }(\theta h{)}^{1/2}\gamma (\kappa ,\theta ,h)∥u-z_{h}^{1}{∥}_{h,V}^{2}+2c_{\varepsilon }h^{-2}∥u-z_{h}^{1}{∥}_{L^{2}(Q)}^{2}+2\varepsilon \;C_{inv,\kappa }∥\sigma_{h}{∥}_{h}^{2}, \end{array}$$

where we used that $\left(\theta h{)}^{1/2}\gamma (\kappa ,\theta ,h\right)>1$, we chose $c_{\varepsilon }>1$, and in the last step we set $C_{inv,k}=1+c_{\mathrm{inv}}\kappa$. Furthermore, using the properties of $a_{h}(\cdot ,\cdot )$ and (29), we have

(43) $$C_{s}∥\sigma_{h}{∥}_{h}^{2}\leq a_{h}(\sigma_{h},\sigma_{h})=a_{h}(u_{h},\sigma_{h})-a_{h}(z_{h}^{1},\sigma_{h})\overset{\left(29\right)}{=}a(u,\sigma_{h})-a(z_{h}^{1},\sigma_{h})=a(u-z_{h}^{1},\sigma_{h}).$$

By replacing $C_{s}=1$ and by choosing $\varepsilon \leq 1/4C_{inv,\kappa }$ in (42), we obtain that

(44) $$\frac{1}{2}∥\sigma_{h}{∥}_{h}^{2}\leq 2c_{\varepsilon }((\theta h{)}^{1/2}\gamma (\kappa ,\theta ,h)∥u-z_{h}^{1}{∥}_{h,V}^{2}+h^{-2}∥u-z_{h}^{1}{∥}_{L^{2}(Q)}^{2}).$$

Now, we can bound the terms in (38). Recalling the definition of $\tilde{\gamma }(\kappa ,\theta ,h)$, inequality (44) immediately implies

(45) $$T_{3}=∥\sigma_{h}{∥}_{h}^{2}\leq C_{\varepsilon }\tilde{\gamma }(\kappa ,\theta ,h)∥u-z_{h}^{1}{∥}_{h,V}^{2}+C_{\varepsilon }h^{-2}∥u-z_{h}^{1}{∥}_{L^{2}(Q)}^{2}.$$

Also, combining (39) and (45), we have that

(46) $$\begin{array}{rl} T_{2} & =\theta h∥\partial_{t}u_{h}^{1}-\partial_{t}z_{h}^{1}{∥}_{L^{2}(Q)}^{2}\leq 2{\tilde{\gamma }}^{2}(\kappa ,\theta ,h)(∥u-z_{h}^{1}{∥}_{h,V}^{2}+∥\sigma_{h}{∥}_{h}^{2})\\ & \leq 2C_{\varepsilon }({\tilde{\gamma }}^{2}(\kappa ,\theta ,h)∥u\;-\;z_{h}^{1}{∥}_{h,V}^{2}\;+\;{\tilde{\gamma }}^{2}(\kappa ,\theta ,h)\tilde{\gamma }(\kappa ,\theta ,h)∥u\;-\;z_{h}^{1}{∥}_{h,V}^{2}\;+\;{\tilde{\gamma }}^{2}(\kappa ,\theta ,h)h^{-2}∥u\;-\;z_{h}^{1}{∥}_{L^{2}(Q)}^{2})\\ & \leq 2C_{\varepsilon }(({\tilde{\gamma }}^{2}(\kappa ,\theta ,h)+{\tilde{\gamma }}^{2}(\kappa ,\theta ,h)\tilde{\gamma }(\kappa ,\theta ,h))∥u-z_{h}^{1}{∥}_{h,V}^{2}+{\tilde{\gamma }}^{2}(\kappa ,\theta ,h)h^{-2}∥u-z_{h}^{1}{∥}_{L^{2}(Q)}^{2}). \end{array}$$

Finally, inserting the bounds (46) and (45) in (38) and using that ${\tilde{\gamma }}^{2}\geq 1$, we obtain

(47) $$\begin{array}{rl} \frac{1}{2}∥u-u_{h}{∥}_{h,*}^{2} & \leq 2C_{\varepsilon }(1+{\tilde{\gamma }}^{2}(\kappa ,\theta ,h)+{\tilde{\gamma }}^{2}(\kappa ,\theta ,h)\tilde{\gamma }(\kappa ,\theta ,h)+\tilde{\gamma }(\kappa ,\theta ,h))∥u-z_{h}{∥}_{h,V}^{2}\\ & \;+2C_{\varepsilon }2{\tilde{\gamma }}^{2}(\kappa ,\theta ,h)h^{-2}∥u-z_{h}^{1}{∥}_{L^{2}(Q)}^{2}. \end{array}$$

Setting $c_{*,V}^{2}=4C_{\varepsilon }$, we can derive estimate (37).

Remark 4.1 Let us consider a fixed $T_{h}(Q)$ and let us recall the forms of $\mu_{1}(\kappa ,\theta ,h)$ and $\mu_{2}(\kappa ,\theta ,h)$ in (37). Then (i) setting a fixed value $\theta =\theta_{c}$, we have $\mu_{1}(\kappa ,\theta_{c},h)∼\theta_{c}h^{-1}+\theta_{c}^{1+1/2}h^{-(1+1/2)}+\theta_{c}^{1/2}h^{1/2}$ and $\mu_{2}(\kappa ,\theta_{c},h)∼$ $h^{-2}+\theta_{c}h^{-3}$, (ii) setting θ≈h, we have that $\mu_{1}(\kappa ,\theta ,h)∼c_{\mathrm{inv}}\kappa$ and $\mu_{2}(\kappa ,\theta ,h)∼h^{-2}$. Note that in the previous cases (i) and (ii), the associated constants depend on $c_{\mathrm{inv}}$ and *κ*.

Below, we recall some approximation estimates of the finite element space. For the proof we refer to [31].

Lemma 4.2. Let s, m be integers such that 0≤m≤1<s and let the space $V_{h0}$ defined in (11). Then for every $v\in V_{0,\underline{0}}$ with $V_{0,\underline{0}}=H_{0,\underline{0}}^{1,1}(Q)\cap H^{s}(Q),$ there exists a linear interpolation operator $\pi_{h}v:V\to V_{h0}$ such that

(48) $$|v-\pi_{h}v{|}_{H^{m}(Q)}\leq c_{\mathrm{intp}}h^{\min(p+1,s)-m}∥v{∥}_{H^{s}(Q)},$$

where $c_{\mathrm{intp}}=c(m,s,Q)$ and *p*=1.

Lemma 4.3. Let the space $V_{h0}$ defined in (11). Let s≥2 be an integer, and let a function $v\in V_{0,\underline{0}}$ with $V_{0,\underline{0}}=H_{0,\underline{0}}^{1,1}(Q)\cap H^{s}(Q)$. There exists a linear interpolation operator $\pi_{h}v:V\to V_{h0}$ such that

(49a) $$∥v-\pi_{h}v{∥}_{L^{2}(\Sigma_{T})}^{2}\leq c_{1}h^{2r-1}∥v{∥}_{H^{s}(Q)}^{2},$$

(49b) $$∥v-\pi_{h}v{∥}_{h,V}^{2}\leq c_{2}h^{2r-1}(\kappa h^{-1}+\theta +\theta^{-1}+1)∥v{∥}_{H^{s}(Q)}^{2},$$

where *r*=2 and $c_{1},c_{2}$ depend on the constants appearing in (17) and in (48), but not on *h* and *v*.

Proof. Introducing the operator $\pi_{h}v$ of Lemma 4.2 and by applying (17) and (48), we have

(50) $$∥u-\pi_{h}v{∥}_{L^{2}(\Sigma_{T})}^{2}\leq 2c_{\mathrm{trac}}^{2}h^{-1}(∥v-\pi_{h}v{∥}_{L^{2}(Q)}^{2})+h^{2}∥\nabla (v-\pi_{h}v){∥}_{L^{2}(Q)}^{2})\leq c_{1}h^{2r-1}∥v{∥}_{H^{s}(Q)}^{2}.$$

In the same way, we have

(51) $$\begin{array}{rl} \kappa ∥\nabla_{x}(v-\pi_{h}v){∥}_{L^{2}(Q)}^{2} & \leq c_{\mathrm{intp}}\kappa h^{2r-2}∥v{∥}_{H^{s}(Q)}^{2},\\ \theta h∥\partial_{t}(v-\pi_{h}v){∥}_{L^{2}(Q)}^{2} & \leq c_{\mathrm{intp}}\theta h^{2r-1}∥v{∥}_{H^{s}(Q)}^{2},\\ (\theta h{)}^{-1}∥(v-\pi_{h}v){∥}_{L^{2}(Q)}^{2} & \leq c_{\mathrm{intp}}\theta^{-1}h^{2r-1}∥v{∥}_{H^{s}(Q)}^{2}, \end{array}$$

Collecting the estimates (50) and (51), we easily obtain

(52) $$∥v-\pi_{h}v{∥}_{h,V}^{2}\leq c_{2}(\kappa h^{2r-2}+\theta h^{2r-1}+\theta^{-1}h^{2r-1}+h^{2r-1})∥v{∥}_{H^{s}(Q)}^{2},$$

which is (49b).

Remark 4.2 The interpolation estimates given in (49) have been derived for linear polynomial spaces, see (11). Analogous estimates can be derived for higher polynomial spaces. In that case we set r=min(p+1,s).

In the proposed scheme (15), the parameter *θ* can control the artificial diffusion terms. Its value can be adjusted according to the needs of the scheme for obtaining optimal approximation properties, see Remark 4.1. Below, we show that if *θ* is close to the grid size *h*, we obtain optimal order of convergence. The value of *θ* can be determined without having to tune it with respect to *κ*. However, in some finite element schemes the value of *θ* must be adjusted with respect to *h* and *κ* for getting optimal rates, e.g. see discussion for the Galerkin/least square methods in [24,33].

Theorem 4.2 **error estimates**

Let *u* be the solution of (7) and let $u_{h}$ be the solution of (15), respectively. Under the Assumption 2.1, the solution $u_{h}$ satisfies the estimate

(53) $$∥u-u_{h}{∥}_{h,*}\leq c_{1}h∥u{∥}_{H^{s}(Q)},\;for\;\theta \approx h,$$

and moreover for any θ>0,

(54) $$(∥u-u_{h}{∥}_{L^{2}(\Sigma_{T})}^{2}+\kappa ∥\nabla_{x}u-\nabla_{x}u_{h}{∥}_{L^{2}(Q)}^{2}+\theta h∥\partial_{t}u_{h}^{b}{∥}_{L^{2}(Q)}^{2}{)}^{1/2}\leq \frac{c_{2}}{\kappa }h∥u{∥}_{H^{s}(Q)},$$

with $c_{1}$ depending on the constants in (37) and in (49) and $c_{2}$ on the constants in (32) and (48).

Proof. The estimate (53) follows directly from (29), (37), Remark (4.1) and (49b). The estimate (54) follows form (32) and (48).

Remark 4.3 In realistic cases, the solutions of parabolic evolution problems may present an anisotropic regularity behavior, for example different regularities properties with respect to time and to space direction. This may result in the case where the contribution of the discretization error in *t* direction can be different than the contribution of the discretzation error in *x* direction. In such cases, it is more appropriate to discretize the problem using anisotropic meshes, using small mesh size in the directions where the solution is less smooth and larger mesh size in the directions where the solution is smoother [34]. This is a topic that we will investigate in a forthcoming paper. A discretization of these type of problems using Isogeometric Analysis methodology is presented in [20].

Remark 4.4 For the sake of simplicity, we have presented the discretization analysis for spaces with degree *p*=1. However, assuming further regularity properties on *u*, we can follow the same analysis and to show optimal estimates for spaces with degree *p*>1, see *Example 2* in Section 5.

## 5. Numerical examples

In this section, we present several numerical examples for validating the theoretical estimates. Although in the analysis, we used linear polynomial spaces, next we perform tests using both linear, (*p*=1), and second order, (*p*=2), polynomial spaces, which are combined with the associated cubic bubble space. We perform tests for $Q\subset R^{d+1}$ with *d*=1 and *d*=2, using triangular and tetrahedral mesh elements correspondingly. One can apply the proposed method for solving problems on $Q\subset R^{d+1}$ with *d*=3. The code that we have at our disposal does not support such calculations yet. Every example has been solved applying several mesh refinement steps with corresponding mesh size $h_{s}=(h_{0}/2^{s}),\;s=1,2,\ldots$. Every $T_{h_{s}}(Q)$ satisfies the properties mentioned in Section 3. We present tables with the asymptotic convergence rates *r* of the error. The convergence rates *r* have been computed by the ratio $r=\ln(e_{s}/e_{s+1})/\ln(h_{s}/h_{s+1})$, where the error $e_{s}:=∥u-u_{h}{∥}_{h,*}$ is computed on $T_{h_{s}}(Q)$. We mention that we use highly smooth solutions, i.e. min(p+1,s)=p+1, and thus the expected values for the rates are *r*=*p*, see (49b) and Remark 4.2. We consider test cases with κ∈{1,0.005}, and we study the behavior of the rates for $\theta =h_{s}$. Lastly, we point out that since the support of a bubble function is restricted to the interior of the element, we eliminate the associated variable from the produced linear system by static condensation.

Example 1: $Q\subset R^{2}$, *p*=1. In the first example, the problem is considered in Q=(0,1)×(0,2). The exact solution is given by the formula

(55) u(x,t)=sin⁡(2πx)sin⁡(πt).

**Figure 2.**
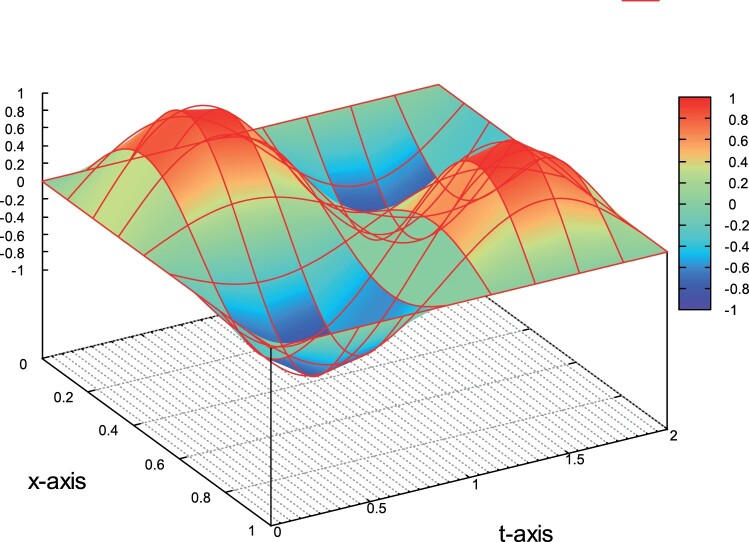
Example 1: The solution *u* on *Q*.

The source function *f* is determined by (55). Note that *u*=0 on Σ and $u_{0}=0$, see (7). In Figure 2, we plot the exact solution on *Q*. We solve the problem using linear polynomials, i.e. *p*=1. We begin by first setting κ=1. The numerical convergence rates for the several levels of the mesh refinement are presented in the second column in Table 1. They are in good agreement with the theoretically predicted estimates given in (53). The numerical solution $u_{h}\in V_{h,b}$ gives optimal convergence rates, i.e. the values of *r* are very close to one for all the refinement steps. Next, we want to investigate the asymptotic behavior of the numerical convergence rates when the value of *κ* is small. We perform the same computations by setting κ=0.005. The associated convergence rates are presented in the last column in Table 1. We observe that for the first mesh levels, the rates *r* are a little higher than the expected value. However, as we move on to the last mesh levels, the values of *r* are close to one, and are in agreement with the values predicted by the theory.

**Table 1.** Example 1: The convergence rates *r*.

$h_{s}$	p=1, κ=1	p=1, κ=0.005
$h_{0}/2^{s}$	Convergence rates *r*	
*s*=1	0.98	1.61
*s*=2	0.99	1.51
*s*=3	0.99	1.20
*s*=4	0.98	1.35
*s*=5	1.00	1.17
*s*=6	0.99	1.05
*s*=7	1.00	1.01

Example 2: $Q\subset R^{2},\;p=2$. In the second example, we consider the problem on Q=(0,1)×(0,1). The exact solution is given by the formula

(56) u(x,t)=sin⁡(2πt)sin⁡(2πx).

**Figure 3.**
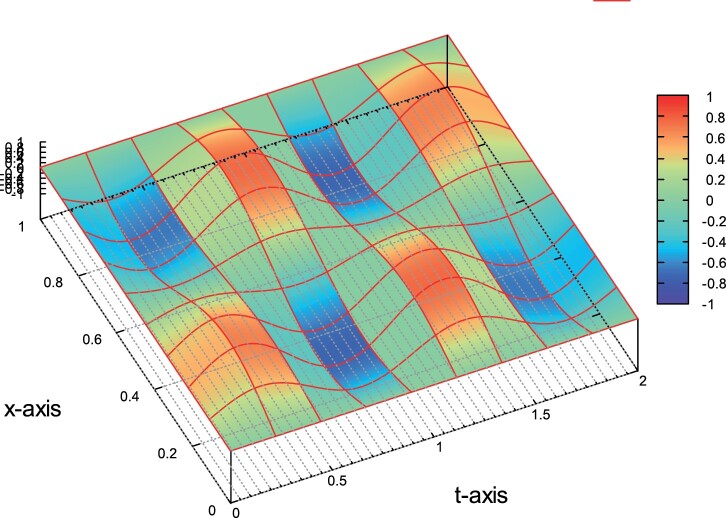
Example 2: The solution *u* on *Q*.

The source function *f* is defined to match the solution in (56). In Figure 3, we plot the exact solution *u* on a relative coarse mesh with *h*=0.25. We solve the problem using second order, i.e. *p*=2, polynomial space. For the first group of computations we set κ=1 and $\theta =h_{s}$. In the second column in Table 2, we show the convergence rates *r*. The values of *r* are approaching the value two, and are the expected rates based (49b) and Remark 4.2. We repeat the same computations setting κ=0.005 and keep the same values for *θ*. The produced rates *r* are shown in the last column in Table 2. We observe that, for the last mesh levels, the rates are approaching the expected value *r*=2. We can see again that the asymptotic convergence behavior of the error is the same for both values of *κ*. Example 3: $Q\subset R^{3}$, *p*=1. In this example, the problem is considered on Q=Ω×(0,1) with $\Omega =(0,1{)}^{2}$. The exact solution is given by the formula

(57) u(x,y,t)=(cos⁡(2π(x−y))−cos⁡(2π(x+y)))sin⁡(2πt).

**Figure 4.**
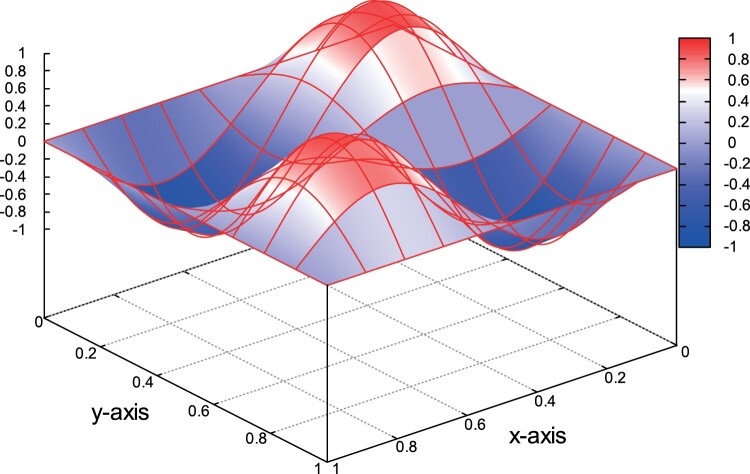
Example 3: The solution *u* on $Q\subset R^{3}$.

Note that *u*=0 on Σ and $u_{0}=0$. The function *f* is determined by (57). In Figure 4, we plot the contours of *u* for *t*=0.8. The problem has been solved on a sequence of meshes, as in the previous tests, using linear polynomial space, i.e. *p*=1. We perform similar computations as before. In the second column in Table 3, we can see the convergence rates for κ=1. The last column in Table 3 shows the rates for κ=0.005. In both cases, the rates are optimal for linear polynomial spaces and are in agreement with the theoretical results in Theorem 4.2.

**Table 2.** Example 2: The convergence rates *r*.

$h_{s}$	p=2,κ=1	p=2, κ=0.005
$h_{0}/2^{s}$	Convergence rates *r*	
*s*=1	1.73	1.83
*s*=2	1.98	2.17
*s*=3	2.01	2.04
*s*=4	2.00	2.05
*s*=5	2.00	2.00
*s*=6	2.00	2.00
*s*=7	2.00	2.00

**Table 3.** Example 3: The convergence rates *r*.

$h_{s}$	p=1, κ=1	p=1, κ=0.005
$h_{0}/2^{s}$	Convergence rates *r*	
*s*=1	1.09	0.60
*s*=2	0.90	1.06
*s*=3	0.92	1.16
*s*=4	0.97	1.15
*s*=5	1.05	1.10

Finally, we can conclude that the proposed bubble stabilization finite element scheme performs well for all the examples. The produced numerical solution gives optimal order of convergence in the $∥\cdot {∥}_{h,*}$-norm, when the problems have smooth solutions.

## 6. Conclusions

In this article, we have proposed and analyzed a bubble stabilized space-time finite element method for solving linear parabolic evolution problems. The construction of the method was based on a space-time variational formulation of the initial PDE problem, which allows the unified space-time discretization by finite element techniques. We presented a discretization error analysis and proved that the method has optimal convergence properties, when θ≈h and the PDE problem has a smooth solution. The convergence properties are not affected by the choice of the value of the diffusion parameter *κ*, which appears in the PDE problem. The theoretical results have been verified by performing several numerical examples. A possible extension of our work here is to combine it with time or space-time mesh adaptivity techniques.
